# Home-based records for poor mothers and children in Afghanistan, a cross sectional population based study

**DOI:** 10.1186/s12889-019-7076-7

**Published:** 2019-06-17

**Authors:** Sayed Ataullah Saeedzai, Iftekhar Sadaat, Zelaikha Anwari, Shafiqullah Hemat, Shakir Hadad, Keiko Osaki, Megumi Asaba, Yohei Ishiguro, Rasuli Mudassir, Jane Machlin Burke, Ariel Higgins-Steele, Khaksar Yousufi, Karen Margaret Edmond

**Affiliations:** 1 Ministry of Public Health, Kabul, Afghanistan; 2 Japan International Cooperation Agency, Tokyo, Japan; 3 Tulane University, New Orleans, USA; 4 Maternal and Child Health Consultant, Beirut, Lebanon; 5 UNICEF, New York, USA; 6 UNICEF, Kabul, Afghanistan

**Keywords:** Home-based records, Personal health records, Mother, Child

## Abstract

**Background:**

No studies have examined distribution, retention and use of maternal and child health (MCH) home-based records (HBRs) in the poorest women in low income countries. Our primary objective was to compare distribution of the new Afghanistan MCH HBR (the MCH handbook) to the poorest women (quintiles 1–2) with the least poor women (quintiles 3–5). Secondary objectives were to assess distribution, retention and use of the handbook across wealth, education, age and parity strata.

**Methods:**

This was a population based cross sectional study set in Kama and Mirbachakot districts of Afghanistan from August 2017 to April 2018. Women were eligible to be part of the study if they had a child born in the last 6 months. Multivariable logistic regression models were constructed to adjust for clustering by district and potential confounders decided a priori (maternal education, maternal age, parity, age of child, sex of child) and to calculate adjusted odds ratios (aOR), 95% confidence intervals (95% CI) and corresponding *p* values. Principal components analysis was used to create the wealth quintiles using standard methods. Wealth categories were ‘poorest’ (quintiles 1,2) and ‘least poor’ (quintiles 3,4,5).

**Results:**

1728/1943 (88.5%) mothers received a handbook. The poorest women (633, 88.8%) had similar odds of receiving a handbook compared to the least poor (990, 91.7%) (aOR 1.26, 95%CI [0.91–1.77], p value 0.165). Education status (aOR 1.03, 95%CI [0.63–1.68], p value 0.903) and age (aOR 1.39, 95%CI [0.68–2.84], p value 0.369) had little effect. Multiparous women (1371, 91.5%) had a higher odds than primiparous women (252, 85.7%) (aOR 1.83, 95%CI [1.16–2.87], p value 0.009). Use of the handbook by health providers and mothers was similar across quintiles. Ten (0.5%) women reported that they received a book but then lost it.

**Conclusions:**

We were able to achieve almost universal coverage of our new MCH HBR in our study area in Afghanistan. The handbook will be scaled up over the next three years across all of Afghanistan and will include close monitoring and assessment of coverage and use by all families.

**Electronic supplementary material:**

The online version of this article (10.1186/s12889-019-7076-7) contains supplementary material, which is available to authorized users.

## Background

There has been much progress within the Afghanistan health system in the last 15 years. However, Afghanistan still has amongst the worst utilisation of health services in the world. 46 percent of mothers use health services for immunisation and less than 15% mothers use services for growth monitoring or promotion [1]. Only 48% of women use health facilities for delivery and 17% use clinics for postnatal care [1]. There is a compelling need for effective interventions that can be used by families in the hardest to reach areas and tools that can empower poor families to use services and take control over children’s and mothers’ health care [2].

Home-based records (HBR) (sometimes called personally controlled, hand held or personal health records) are widely used globally, including in remote areas. They have many different forms including paper and electronic [3, 4]. Common features of HBRs are ownership by the family not the health service and that they are kept at home. Families are requested to bring the HBR or to provide electronic access to their HBR at health visits [3, 4].

Some countries use single ‘standalone’ HBRs (e.g. vaccination cards, growth monitoring cards, antenatal cards) [4–12]. Advantages include simplicity and low cost. However, many countries use integrated (also called combined) maternal and child health (MCH) HBRs instead [6]. Integrated MCH HBRs include health promotion messages and health records across antenatal care (ANC), delivery, birth registration, postnatal care (PNC), vaccinations, nutritional and early childhood development services [3, 5, 7]. In mid 2018, after a series of detailed systematic reviews of MCH HBRs, the World Health Organization (WHO) concluded that MCH-HBRs can improve communication and continuum between health service providers and can improve the communication of important health information to families [3, 11]. However, no studies appear to have been published which have assessed distribution, retention, and use of integrated HBRs, especially in the poorest families in fragile states such as Afghanistan who need them most.

Since January 2016, the Ministry of Public Health in Afghanistan (MoPH), Japan International Cooperation Agency (JICA), United Nations Children’s Fund (UNICEF), WHO and other partners have been working together to develop Afghanistan’s first ever integrated HBR for maternal and child health (MCH) care (called the MCH handbook). In the first pilot phase two districts were purposively chosen for implementation and evaluation.

Thus, our primary objective was to compare the distribution of the new Afghanistan MCH handbook between poor and less poor women in the two pilot districts. Secondary objectives were to assess retention and to understand if there were important differentials in distribution across specific strata (maternal education, maternal age, parity). We also assessed if there was variation in how health care providers and mothers utilised the handbook across wealth quintiles.

## Methods

### Design

This was a cross sectional, population based study of the implementation of the MCH handbook into routine service delivery in two pilot districts (Kama and Mirbachakot) of Afghanistan. It was conducted over a nine month period from August 2017 to April 2018.

### Study population

As part of routine service delivery, all women were eligible to receive a handbook if they were pregnant or had a child aged less than 24 months.

Women were eligible to be a respondent in the cross sectional evaluation study if they were married, aged between 15 and 49, had a child born in the past six months and lived permanently in Kama or Mirbachakot district from August 2017 to April 2018.

We restricted the respondent population for the cross sectional evaluation to women with a child less than six months of age because the handbooks had only been distributed for nine months and we wanted to capture as many women with handbooks as possible. There were no exclusions.

### Study setting

Afghanistan is a mountainous landlocked country with deteriorating security and increasing levels of conflict over the last five years [13, 14]. Kama and Mirbachakot districts were purposively chosen for this study to provide health system, access, socio economic and conflict characteristics that are representative of many districts in Afghanistan. Table 1 displays district profile data.

**Table 1:** Characteristics of Mirbachakot and Kama districts of Afghanistan from August 2017 to April 2018

	Mirbachakot	Kama
Population^a^		
Total population of district	97,631	43,164
Number of women of reproductive age	19,526	8633
Number of children under 1 year	3905	1727
Access		
Mountainous district^b^	No	No
Remoteness^c^	Yes	Yes
Distance in kms from provincial capital	40	50
District security risk^d^	Medium	Medium
Sociodemographics^a^		
% reproductive age women in lowest wealth quintile	16.9%	20.1%
% reproductive age women with no education	49.1%	68.5%
% reproductive age women with any contraception use	26.5%	13.3%
% reproductive age women who report difficulties accessing health care	50.8%	79.7%
Health services^e^		
Total number of fixed health facilities (Sub health centre, basic health centre, comprehensive health centre, district hospital)	9	5
Total population per fixed health facilities	10,848	8638
Number of district hospitals	1	1
Number of comprehensive health centres	1	1
Number of basic health centres	3	3
Number of sub health centres	4	0
Number of health posts	70	38
Number of doctors	18	7
Number of midwives	24	11
Number of vaccinators	23	14
Number of nutrition counsellors	4	1
Number of community health workers	139	76

^a^ Afghanistan Demographic and Health Survey (AfDHS 2015) [1]

^b^ Mountainous = More than 1800 km elevation at highest point of district. SDES 2016 [14]

^c^ Remote = District centre more than 2 h by any form of transport from provincial capital. Afghanistan Social Demographic and Economic Survey (SDES 2016) [14]

^d^ Security risk = Use of armed force between warring parties in a conflict dyad, state-based or non-state, resulting in deaths. 25 deaths or less in the previous 12 months is categorised as low intensity security risk, 25–100 is categorised as moderate intensity security risk and 100+ is categorised as high intensity security risk. World Bank 2016 [13]

^e^ Afghanistan Health Management Information System (HMIS 2017) [15]

There are functional hospital and health centres throughout Afghanistan though services can be forced to close by anti-government elements [13]. The primary health care system includes accredited nurses and doctors who work in subcentres [SHC], basic health centres [BHC], comprehensive health centres [CHC] and national, regional, provincial and district hospitals [15–17]. The doctors and midwives provide all medical tests, medical examinations, ANC, PNC and birthing services in the health facilities. The primary health care system also includes volunteer CHWs who work in ‘health posts’ (usually their own home in their own villages). CHWs are also trained to provide family planning, maternal and child health promotion education, basic medicines (e.g. oral contraceptives, iron and folic acid, antibiotics, oral rehydration solution) and to refer mothers and children with significant illness and danger signs. CHWs are not accredited to provide medical or birthing services which must be provided by accredited doctors and midwives. The CHWs are supervised by paid community health supervisors who provide monthly village based supportive supervision [16].

### MCH handbook implementation

The new Afghanistan MCH handbook records all stages of maternal, newborn, and child health from antenatal care to delivery, birth registration, postnatal care, nutrition counselling, child vaccinations, development, hygiene, growth monitoring, early child development and family planning. It is pictorial with illustrated health promotion messages and has space for recording ANC, delivery, PNC, immunisation and growth monitoring services. The handbook was printed in Dari and Pashto (the two main local languages). All the health records, illustrations and health promotion messages were directly replicated from existing materials that had been already been focus group tested in Dari and Pashto.

MCH handbook implementation had six components (i) development of the handbook (ie combination of all the standalone materials into the handbook); (ii) revisions to registers, tally sheets, and stock cards; (iii) development of training materials; (iv) training of health care providers; (v) distribution of the handbook by the usual MoPH supply chain (ie the same supply chain that was used for the standalone vaccination and ANC cards); and (vi) monitoring and supervision at point of service.

Health provider training focused on the need to: (i) provide the MCH handbook to all pregnant women and families of children under 24 months; (ii) explain the health promotion and record keeping components of the book to families; and (iii) remind families to bring the MCH handbook to all health visits. A total of 150 personnel, both care providers and management staff, were trained in the use of the MCH handbook, i.e. all doctors, midwives, nurses, vaccinators, and nutrition counsellors who work in all eight public health facilities (i.e. 2 district hospitals, 6 primary health centers) in the study area, and relevant provincial and district officers.

The MCH handbook was distributed to families in the two pilot districts (Kama and Mirbachakot) from August 2017 and continues to the present time.

Women received a handbook if they were pregnant or had a child born in the past 24 months. They were able to receive the book when they accessed MCH services (e.g. ANC, facility delivery, PNC, immunisation and growth monitoring). For an estimated 16,086 pregnant women and children aged less than 24 months, 25,500 handbooks were prepared and 21,500 handbooks were distributed by the end of June 2018.

### Data collection

For data collection, we randomly selected households to be visited in each district using a standard two stage sampling method with probability of selection proportional to size (i.e. random selection of villages followed by random selection of households). The basic sampling frame was obtained from the Health Management Information System (HMIS) which listed the names of the districts, their villages and their population size. Use of the same cooking hearth was used to define a household. Households were selected within the villages using the ‘random walk’ method [18].

Questionnaire data were collected from eligible mothers during household visits by trained female field workers using a standardised electronic structured questionnaire (Additional file 1). Data were collected on socio economic status, receipt of any HBR and date of receipt. Mothers were also asked if they received any explanation about HBRs at the time of receipt. Mothers were asked if they still had the HBRs and to show them to the interviewer. Mothers were asked if they looked at the illustrations and records and if they would show the HBR to their friends and family. The interviewer checked completion of all data fields in the HBR including child’s name and date of birth. Senior supervisors reviewed each questionnaire and 10% of mothers were revisited for data checking.

### Data analysis

Principal components analysis was used to create wealth quintiles using standard methods [19]. Wealth categories were defined as ‘poorest’ (quintiles 1,2) and ‘least poor’ (quintiles 3,4,5). The primary outcome measure was the proportion of ‘poor women’ (i.e. women in quintiles 1,2) women who reported receiving a MCH handbook in the study area compared to ‘least poor women’ (i.e. women in quintiles 3,4,5). We calculated that we required a sample size of 1500 women to provide 90% power at a 5% significance level to assess effects on the primary outcome assuming a HBR distribution rate of 50–60% and a 10% difference between poor and least poor families [1]. This sample size also provided sufficient power for analysis of secondary outcomes. Multivariable logistic regression models were constructed to adjust for clustering by district and potential confounders decided a priori (maternal education, maternal age, parity, age of child when they received the handbook, sex of child) and to calculate adjusted odds ratios (aOR), 95% confidence intervals (95% CI) and corresponding *p* values.

Stata version 15.1 was used for all analyses.

## Results

Characteristics of the districts were similar (Table 1) including security risk, remoteness, wealth and facility density. 1943 out of 2045 (95%) eligible mothers agreed to participate in the study (1094 Kama, 849 Mirbachakot) (Table 2). Characteristics of women were similar in the two districts. 78.5% (1417) of women had no education, 33.7% (654) were under 25 years of age and 15.7% (304) were primipara. Due to an electronic data collection error, socio economic data were not collected on 7.2% (139) of women. There were no obvious differences in socio demographic variables between women with and without wealth data (Table 3).

**Table 2:** Characteristics of MCH handbook respondents in Mirbachakot and Kama districts of Afghanistan from August 2017 to April 2018

	Mirbachakot (*n* = 849)	Kama (*n* = 1094)	Total (*n* = 1943)
Wealth categories^a^			
Poorest	280 (32.9%)	442 (40.4%)	722 (37.3%)
Least poor	567 (67.7%)	515 (47.1%)	1082 (55.7%)
Not known	2 (0.3%)	137 (12.5%)	139 (7.2%)
Wealth quintile^b^			
Poorest [1]	137 (16.1%)	224 (20.5%)	361 (18.7%)
2	143 (16.8%)	218 (19.9%)	361 (18.6%)
3	169 (19.9%)	192 (17.6%)	361 (18.6%)
4	246 (29.0%)	115 (10.5%)	361 (18.6%)
Least poor [5]	152 (17.9%)	208 (19.0%)	360 (18.5%)
Not known	2 (0.24%)	137 (12.5%)	139 (7.2%)
Maternal education			
No education	597 (70.3%)	927 (84.7%)	1524 (78.4%)
Primary	101 (11.9%)	48 (4.4%)	149 (7.7%)
Secondary+	107 (12.6%)	63 (5.8%)	170 (8.8%)
Not known	44 (5.2%)	56 (5.1%)	100 (5.2%)
Maternal age			
16-19y	40 (4.7%)	34 (3.1%)	74 (3.8%)
20-24y	279 (32.9%)	301 (27.5%)	580 (29.9%)
25-29y	271 (31.9%)	269 (24.6%)	540 (27.8%)
30-34y	171 (20.1%)	260 (23.8%)	431 (22.2%)
35 + y	88 (10.4%)	230 (21.0%)	318 (16.4%)
Parity			
1	162 (19.1%)	142 (13.0%)	304 (15.7%)
2–6	588 (69.3%)	702 (64.2%)	1290 (66.4%)
7+	99 (11.7%)	250 (22.9%)	349 (18.0%)
Age of child			
< 1 m	101 (11.9%)	4 (0.37%)	105 (5.4%)
1 m	151 (17.8%)	118 (10.8%)	269 (13.8%)
2 m	110 (13.0%)	101 (9.2%)	211 (10.9%)
3 m	106 (12.5%)	140 (12.8%)	246 (12.7%)
4 m	84 (9.9%)	145 (13.3%)	229 (11.8%)
5 m	85 (10.0%)	165 (15.1%)	250 (12.9%)
6 m	212 (25.0%)	421 (38.5%)	633 (32.6%)
Sex of child			
Female	412 (48.5%)	529 (48.4%)	941 (48.4%)
Male	437 (51.5%)	565 (51.7%)	1002 (51.6%)

*MCH* maternal and child health

^a^Wealth category = poorest = quintiles 1,2; least poor = quintiles 3,4,5

^b^ Quintiles calculated using principal components analysis [19]

**Table 3:** Characteristics of MCH handbook respondents according to maternal wealth status in Mirbachakot and Kama districts of Afghanistan from August 2017 to April 2018

	Poorest	Least poor	Total in wealth status known	Wealth status not known	Total
	(*n* = 722)	(*n* = 1082)	(*n* = 1804)	(*n* = 139)	(*n* = 1943)
Wealth quintile^a^					
Lowest quintile 1	361 (50.0%)	0 (0.0%)	361 (20.0%)	0 (0.0%)	361 (18.6%)
2	361 (50.0%)	0 (0.0%)	361 (20.0%)	0 (0.0%)	361 (18.6%)
3	0 (0.0%)	361 (33.6%)	361 (20.0%)	0 (0.0%)	361 (18.6%)
4	0 (0.0%)	361 (33.6%)	361 (20.0%)	0 (0.0%)	361 (18.6%)
Highest quintile 5	0 (0.0%)	360 (33.3%)	360 (19.8%)	0 (0.0%)	360 (18.5%)
Not known	0 (0.0%)	0 (0.0%)	0 (0.0%)	139 (100.0%)	139 (7.2%)
Maternal education					
No education	612 (84.8%)	805 (74.4%)	1417 (78.5%)	107 (77.0%)	1524 (78.4%)
Primary	39 (5.4%)	103 (9.5%)	142 (7.9%)	7 (5.0%)	149 (7.7%)
Secondary+	37 (5.1%)	121 (11.2%)	158 (8.8%)	12 (8.6%)	170 (8.8%)
Not known	34 (4.7%)	53 (4.9%)	87 (4.8%)	13 (9.4%)	100 (5.2%)
Maternal age					
16-19y	32 (4.4%)	42 (3.9%)	74 (4.1%)	0 (0.0%)	74 (3.8%)
20-24y	199 (27.6%)	355 (32.8%)	554 (30.7%)	26 (18.7%)	580 (29.9%)
25-29y	189 (26.2%)	319 (29.5%)	508 (28.2%)	32 (23.0%)	540 (27.8%)
30-34y	174 (24.1%)	205 (19.0%)	379 (21.0%)	52 (37.4%)	431 (22.2%)
35 + y	128 (17.7%)	161 (14.9%)	289 (16.0%)	29 (20.9%)	318 (16.4%)
Parity					
1	145 (20.1%)	152 (14.1%)	197 (10.9%)	7 (5.0%)	304 (15.7%)
2–6	469 (65.0%)	732 (67.7%)	1201 (66.6%)	89 (64.0%)	1290 (66.4%)
7+	108 (15.0%)	198 (18.3%)	306 (17.0%)	43 (30.9%)	349 (18.0%)
Age of child					
< 1 m	41 (5.7%)	64 (5.9%)	105 (5.8%)	0 (0.0%)	105 (5.4%)
1 m	106 (14.7%)	144 (13.3%)	250 (13.9%)	19 (13.7%)	269 (13.8%)
2 m	92 (12.7%)	108 (10.0%)	200 (11.1%)	11 (7.9%)	211 (10.9%)
3 m	87 (12.1%)	138 (12.8%)	225 (12.5%)	21 (15.1%)	246 (12.7%)
4 m	68 (9.4%)	142 (13.1%)	210 (11.6%)	19 (13.7%)	229 (11.8%)
5 m	95 (13.2%)	135 (12.9%)	230 (12.7%)	20 (14.4%)	250 (12.9%)
6 m	233 (32.3%)	351 (32.4%)	584 (32.4%)	49 (35.3%)	633 (32.6%)
Sex of child					
Female	335 (46.4%)	517 (47.8%)	852 (47.2%)	89 (64.0%)	941 (48.4%)
Male	387 (53.6%)	565 (52.2%)	952 (52.8%)	50 (36.0%)	1002 (51.6%)

*MCH* Maternal and child health

^a^Wealth category = poorest = quintiles 1,2; least poor = quintiles 3,4,5

1728 (88.5%) of the 1943 women who agreed to participate in the study received a handbook. Women reported that they owned their handbooks for a mean 6.1 (sd 2.1) months (Table 4). Ten women (0.5%) reported that they had received a book but then lost it (Table 4, Additional file 2). Seven of these women owned their handbook for 4 months or longer. 32 women (1.6%) received more than one book (Table 4).

**Table 4:** MCH handbook distribution and use according to maternal wealth status in Mirbachakot and Kama districts of Afghanistan from August 2017 to April 2018

	Poorest^a^	Least poor^a^	Total in wealth status known	Wealth status not known	Total
MCH handbook distribution	(*n* = 722)	(*n* = 1082)	(*n* = 1804)	(*n* = 139)	(*n* = 1943)
Received and retained	632 (87.5%)	982 (90.8%)	1614 (89.5%)	104 (74.8%)	1718 (88.4%)
Received but lost	1 (0.1%)	8 (0.7%)	9 (0.5%)	1 (0.7%)	10 (0.5%)
Did not receive	80 (11.1%)	90 (8.3%)	170 (9.4%)	34 (24.5%)	204 (10.5%)
Not known	9 (1.3%)	2 (0.2%)	11 (0.6%)	0 (0%)	11 (0.6%)
Number of MCH handbooks received	(*n* = 722)	(*n* = 1082)	(*n* = 1804)	(*n* = 139)	(*n* = 1943)
1	620 (85.9%)	962 (88.9%)	1582 (87.7%)	103 (74.1%)	1685 (86.7%)
2	8 (1.1%)	21 (1.9%)	29 (1.6%)	1 (0.7%)	30 (1.5%)
3	1 (0.1%)	1 (0.1%)	2 (0.1%)	0 (0.0%)	2 (0.1%)
Did not receive	13 (1.8%)	8 (0.7%)	21 (1.2%)	1 (0.7%)	22 (1.1%)
Not known	80 (11.1%)	90 (8.3%)	170 (9.4%)	34 (24.5%)	204 (10.5%)
Months owned MCH handbook	(*n* = 632)	(*n* = 982)	(*n* = 1614)	(*n* = 104)	(*n* = 1718)^b^
1 month	24 (3.8%)	29 (3.0%)	53 (3.3%)	2 (1.9%)	55 (3.2%)
2 months	48 (7.6%)	58 (5.9%)	106 (6.6%)	5 (4.8%)	111 (6.5%)
3 months	24 (3.8%)	30 (3.1%)	54 (3.4%)	14 (13.5%)	68 (4.0%)
4 months	45 (7.1%)	57 (5.8%)	102 (6.3%)	16 (15.4%)	118 (6.9%)
5 months	63 (10.0%)	92 (9.4%)	155 (9.6%)	12 (11.5%)	167 (9.7%)
6 months	81 (12.8%)	123 (12.5%)	204 (12.6%)	6 (5.8%)	210 (12.2%)
7 months	85 (13.5%)	193 (19.7%)	278 (17.2%)	13 (12.5%)	291 (16.9%)
8+ months	189 (29.9%)	348 (35.4%)	537 (33.3%)	27 (26.0%)	564 (32.8%)
Not known	73 (11.6%)	52 (5.3%)	125 (7.7%)	9 (8.7%)	134 (7.8%)
Mean (sd)	5.87 (2.2)	6.23 (2.01)	6.10 (2.09)	5.48 (2.13)	6.06 (2.09)
Median (iqr)	6 (4–8)	7 (5–8)	7 (5–8)	5 (4–8)	7 (5–8)
Completion of records by health provider	(n = 632)	(n = 982)	(n = 1614)	(n = 104)	(n = 1718)^a^
Name of child	574 (90.8%)	787 (80.1%)	1361 (84.3%)	100 (96.2%)	1426 (83.0%)
Date of birth of child	564 (89.2%)	762 (77.6%)	1326 (82.2%)	102 (98.1%)	1463 (85.2%)
Any ANC visits	501 (79.3%)	607 (61.8%)	1108 (68.7%)	90 (86.5%)	1198 (69.7%)
Any PNC visits	496 (78.5%)	623 (63.4%)	1119 (69.0%)	79 (76.0%)	1198 (69.7%)
Birth polio vaccine	605 (95.7%)	946 (96.3%)	1551 (96.0%)	103 (99.0%)	1654 (96.3%)
First pentavalent vaccine	569 (90.0%)	800 (81.5%)	1369 (84.8%)	92 (88.5%)	1461 (85.0%)
Birth weight	471 (74.5%)	556 (56.6%)	1027 (63.6%)	97 (93.3%)	1124 (65.4%)
Growth chart curve	480 (76.0%)	590 (60.1%)	1070 (66.3%)	101 (97.1%)	1171 (68.2%)
Information communicated to mother by health provider	(*n* = 632)	(*n* = 982)	(*n* = 1614)	(*n* = 104)	(*n* = 1718)^b^
About purpose of the handbook	600 (94.9%)	890 (90.6%)	1490 (92.3%)	93 (89.4%)	1583 (92.1%)
That the mother should bring the handbook with her to all health visits	309 (48.9%)	472 (48.1%)	781 (48.4%)	75 (72.1%)	856 (49.8)
Use of MCH handbook by mother	(*n* = 632)	(*n* = 982)	(*n* = 1614)	(*n* = 104)	(*n* = 1718)^b^
Read the health care messages	248 (39.2%)	561 (57.1%)	809 (50.1%)	38 (36.5%)	847 (49.3%)
Reviewed own or child’s health records	329 (52.1%)	501 (51.0%)	830 (51.4%)	82 (78.9%)	912 (53.1%)
Showed to family members, friends or neighbours	419 (66.3%)	738 (75.2%)	1157 (71.7%)	102 (98.1%)	1259 (73.3%)
Took to visits with health care workers	462 (73.1%)	813 (82.8%)	1275 (79.0%)	96 (92.3%)	1371 (79.8%)
Looked at the illustrations	487 (77.1%)	798 (81.3%)	1285 (79.6%)	98 (94.2%)	1383 (80.5%)
Used for at least one specific purpose as above	521 (82.4%)	939 (95.6%)	1460 (90.5%)	104 (100.0%)	1564 (91.0%)

*MCH* Maternal and child health, *ANC* antenatal care, *PNC* postnatal care

^a^Wealth category = poorest = quintiles 1,2; least poor = quintiles 3,4,5

^b^ In all 1718 women who received and retained the handbook

Distribution of handbooks was not associated with wealth status (Table 5). The poorest women (quintiles 1–2) (633, 88.8%) had similar odds of receiving a book compared to the least poor women (990, 91.7%) (quintiles 3–5) (adjusted odds ratio [aOR] 1.26, 95%CI 0.91–1.77), *p* value 0.165) (Table 5).

**Table 5:** Association between socio demographic variables and MCH handbook distribution in Mirbachakot and Kama districts of Afghanistan from August 2017 to April 2018

	Frequency			Crude analysis		Adjusted analysis^a^	
	Total	Did not receive	Received	OR (95% CI)	*p* value	aOR (95% CI)	*p* value
	*n* = 1793^b^	*n* = 170	*n* = 1623				
Wealth groupings							
Poorest	713 (100%)	80 (11.2%)	633 (88.8%)	1.00		1.00	
Least poor	1080 (100%)	90 (8.3%)	990 (91.7%)	1.39 (1.01–1.91)	0.042	1.26 (0.91–1.77)	0.165
Wealth quintile							
1 (poorest)	356 (100%)	45 (12.6%)	311 (87.4%)	1.00		1.00	
2	357 (100%)	35 (9.8%)	322 (90.2%)	1.33 (0.83–2.13)	0.231	1.34 (0.83–2.16)	0.239
3	360 (100%)	28 (7.8%)	332 (92.2%)	1.72 (1.04–2.82)	0.033	1.51 (0.90–2.52)	0.115
4	360 (100%)	31 (8.6%)	329 (91.4%)	1.54 (0.95–2.49)	0.082	1.35 (0.81–2.26)	0.247
5 (least poor)	360 (100%)	31 (8.6%)	329 (91.4%)	1.54 (0.95–2.49)	0.082	1.51 (0.91–2.50)	0.113
Maternal education							
No education	1409 (100%)	140 (9.9%)	1269 (90.1%)	1.00		1.00	
Primary	141 (%)	12 (8.5%)	129 (91.5%)	1.19 (0.64–2.20)	0.588	0 .87 (0.45–1.67)	0.677
Secondary+	156 (%)	13 (8.3%)	143 (91.7%)	1.21 (0.67–2.20)	0.523	1.23 (0.65–2.33)	0.529
Maternal age							
16-19y	74 (100%)	11 (14.9%)	63 (85.1%)	1.00		1.00	
20-24y	546 (100%)	55 (10.1%)	491 (89.9%)	1.56 (0.78–3.13)	0.213	1.40 (0.67–2.91)	0.373
25-29y	508 (100%)	34 (6.7%)	474 (93.3%)	2.43 (1.17–5.05)	0.017	1.71 (0.77–3.81)	0.188
30-34y	378 (100%)	40 (10.6%)	338 (89.4%)	1.48 (0.72–3.03)	0.289	1.06 (0.47–2.40)	0.879
35 + y	287 (100%)	30 (10.5%)	257 (89.6%)	1.50 (0.71–3.15)	0.289	1.09 (0.47–2.55)	0.841
Parity							
1	294 (100%)	42 (14.3%)	252 (85.7%)	1.00		1.00	
2–6	1195 (100%)	102 (8.5%)	1093 (91.5%)	1.79 (1.22–2.62)	0.003	1.81 (0.15–2.85)	0.011
7+	304 (100%)	26 (8.6%)	278 (91.5%)	1.78 1.06–2.99)	0.029	2.05 (1.09–3.88)	0.027
Sex of child							
Female	844 (100%)	96 (11.4%)	748 (88.6%)	1.00		1.00	
Male	949 (100%)	74 (7.8%)	875 (92.2%)	1.52 (1.10–2.09)	0.010	1.53 (1.10–2.12)	0.011
Age of child							
< 1 m	104 (100%)	16 (15.4%)	88 (84.6%)	1.00		1.00	
1 m	247 (100%)	17 (6.9%)	230 (93.1%)	2.46 (1.19–5.08)	0.015	2.68 (1.27–5.67)	0.010
2 m	198 (100%)	9 (4.6%)	189 (95.5%)	3.82 (1.62–8.98)	0.002	4.20 (1.74–10.13)	0.001
3 m	225 (100%)	8 (3.6%)	217 (96.4%)	4.93 (2.04–11.9)	0.000	5.35 (2.04–14.00)	0.001
4 m	209 (100%)	13 (6.2%)	196 (93.8%)	2.74 (1.26–5.94)	0.011	2.93 (1.30–6.62)	0.010
5 m	230 (100%)	34 (14.8%)	196 (85.2%)	1.05 (0.55–2.00)	0.886	1.18 (0.59–2.35)	0.644
6 m+	580 (100%)	73 (12.6%)	507 (87.4%)	1.26 (0.70–2.27)	0.436	1.37 (0.72–2.59)	0.336
District							
Kama	957 (100%)	102 (10.7%)	855 (89.3%)	1.00		1.00	
Mirbachakot	836 (100%)	68 (8.1%)	768 (91.9%)	1.35 (0.98–1.86)	0.070	1.32 (0.91–1.90)	0.144

*MCH* maternal and child health

Wealth category = poorest = quintiles 1,2; least poor = quintiles 3,4,5

*OR* odds ratio, *aOR* adjusted odds ratio

^a^ Adjusted for quintile, maternal education, maternal age, parity, age of child, sex of child

^b^ In women who had known status of receipt of the MCH handbook and also had known socio economic data (*n* = 1793)

Distribution of the handbooks was not associated with maternal education level (Table 5). Women with no education (1269, 90.1%) had similar odds of receiving a book compared to women who received at least primary (272, 91.5%) and secondary (143 (91.7%) level education (aOR 1.03, 95%CI [0.63–1.68], p value 0.903). Distribution of the handbooks was also not associated with maternal age (Table 5). Women aged 16–19 years (63, 85.1%) had similar odds of receiving a book compared to older women aged 20 years and above (1560, 90.8%) (aOR 1.39, 95%CI [0.68–2.84], p value 0.369).

However, there were small but significant differences in some other variables. Multiparous women (1371, 91.5%) had a higher odds of receiving a handbook than primiparous women (252, 85.7%) (aOR 1.83, 95%CI [1.16–2.87], p value 0.009). Also mothers with male infants had a higher odds of receiving a handbook than mothers with female infants (aOR 1.53, 95%CI [1.10–2.12], p value 0.011) (Table 5).

The majority of health records were completed ranging from vaccination (birth dose polio vaccine [96.0%, 1551] to birth weight [63.6%, 1027] (Table 4). 1490 (92.3%) mothers reported that the health provider explained the purpose of the handbook. However, only 781 (48.4%) mothers reported that the health provider said she should bring the handbook to all health visits. Similar proportions were reported for poor and least poor mothers (Table 4).

1564 (91.0%) mothers reported that they used the handbook for at least one specific purpose. The most common reason was to look at the illustrations (80.5%, 1383). 1371 (79.8%) reported that they took the handbook to all health visits. In contrast, only 847 (49.3%) used the book to read health care messages and 912 (53.1%) used the book to review their own or their child’s health records. Use was slightly lower (82.4%, 521/632) in poor compared to least poor mothers (95.6%, 939/982) (Table 4). Use appeared similar in non educated (90.2% 1210/1341) and educated (93.7% 267/285) mothers.

## Discussion

We were able to achieve almost universal coverage of the integrated MCH HBR in our study area in Afghanistan. We achieved 89% distribution in the poorest and 92% distribution in the least poor mothers. Mothers with no education (90%), young mothers (85%) and primiparous (85%) mothers also had high coverage. Retention of handbooks was encouragingly high (99%), only ten women misplaced or lost their books.

To our knowledge, this is the first study of the distribution, retention and use of HBRs in the poorest women in both low and high income countries and also the first from a conflict affected country. In particular, there have been no studies that assessed effects of integrated HBRs on distribution, retention (i.e. mothers not ‘losing the HBRs’) and use at the ‘point of care’ (i.e. mothers remembering to bring the records to the clinic or hospital) [3]. There have also been no studies from fragile countries and no studies which included the poorest families who need them most.

Twenty seven quasi- and non- randomised studies implemented in high [8], middle [5] and low [2] income countries have examined the effectiveness of MCH HBRs [3, 20]. Most of these studies reported that families who received HBRs had better use of MCH services compared to families who received usual care services without HBRs. Impacts included improved immunization, infant feeding, nutrition and early child development services and practices [3]. Improved information sharing between health care providers and families was also reported [3]. However, there have been only two randomised controlled trials which have reported on availability of MCH records at the point of care (i.e. mothers not losing their books and remembering to bring their books to the clinic) [20–23]. These studies reported that availability of MCH records was 20–30% higher in women who received HBRs compared to women who received usual care services without HBRs. However, the individual and pooled results were not statistically significant (pooled RR 0.38, 95% CI 0.04 to 3.84) and both studies were conducted in high income countries [20–23].

Cross-sectional Demographic and Health Survey (DHS) studies, have also been published [9]. Low rates (30–60%) of retention of HBRs were reported across the 180 DHS surveys that were assessed. It is encouraging that retention in our study was higher (88% than those DHS studies and the recent Afghanistan DHS report of 56% in 2015) [1]. However, it is important to note that our study included mothers of children under 6 months while the DHS surveys include children aged 12–23 months only. In addition, our study was conducted only 9 months after distribution commenced and longer term follow up of our study is needed. All 10 women who lost their book in our study had held the book for over 4 months.

It is encouraging that we detected no differential in distribution or retention by wealth quintile. DHS data indicate a ‘dose response’ in vaccine card retention i.e. as poverty level increases the proportion of families with who retain vaccination cards decrease [1]. Indeed, the latest Afghanistan DHS reported vaccination card retention of 49% in the poorest and 69% in the least poor quintile, compared to 88% in the poorest and 91% in the least poor quintile in our study [1]. No other studies, to our knowledge, have assessed distribution and retention of HBRs in the poorest families.

It is also encouraging that distribution was 90% in women with no education and 85% in young women aged 16–19 years. We found that distribution was 1.8 fold lower in primiparous (86%) than multiparous women (92%); and mothers with female infants (89%) were 1.5 times less likely to receive a handbook than mothers with male infants (92%). However, the absolute differences were small in these analyses and the findings could be due to chance alone. We will monitor these issues closely during our scale up phase through to 2021.

Completion of demographic information and vaccination records by health providers was much better than completion of growth monitoring and midwifery care. 96% completed the polio birth dose, 80% completed name and date of birth of the child and 92% of health providers explained the purpose of the handbook. However, only 64% completed birth weight and 69% completed ANC records. Similar findings were observed for both poor and least poor mothers. In addition, only 48% of health providers told the mothers to bring the handbook to health visits. It is well known that health providers use HBRs poorly in both high and low income countries [3, 8, 12, 24]; but our findings still show that there are many missed opportunities for providing health care for mothers and children who live in our study area. Improved training of health care providers on all parts of MCH handbook service provision is essential.

Ninety percent of mothers reported that they used the MCH handbook for at least one specific purpose. Given the low rates of literacy it was not surprising that the most common reason was to look at illustrations (80%) and only 50% read the health care messages. It is encouraging that 78% took the handbook to health visits and 73% showed it to family and friends. Similar utilisation was reported by poor and non educated mothers.

Interestingly, similar findings are reported from qualitative studies [11, 21]. These qualitative studies indicate that integrated HBRs are more highly valued and ‘remembered’ by mothers and health service providers than ‘stand alone’ records because the same source of health information is repeatedly used across the antenatal, postnatal and infant life course [11, 21].

There were many challenges in implementing our study due to conflict and closure of health facilities. However, in Afghanistan there is a robust supply chain for vaccination, ANC, PNC, family planning and nutritional commodities and a well functioning (mainly paper based) HMIS [16, 25]. This system was used successfully for the distribution of the MCH handbook in this pilot study and we will continue to use these tools during the scale up phase.

Our study did have limitations. it was cross sectional and our districts were purposively chosen to provide generalisability in Afghanistan. All health service utilization data were self-reported and were not verified due to logistic reasons. However any misclassification should have been non-differential across wealth groupings. We were also only able to assess retention and use over 9 months and longer term follow up is needed. Important strengths included our population based implementation and evaluation and our large sample size. We had a high response rate and our well trained female field workers were able to interview mothers within the household. Retention and data recording were directly verified by the field work team.

Many MCH services including vaccination, ANC and growth monitoring are well known to have the poorest coverage in the poorest families [26], including in Afghanistan [27–29]. Thus, it is encouraging that the MCH handbook, an integrated MCH HBR, could be implemented in the complex environment of rural Afghanistan and that it could reach high numbers of poor mothers. It also is encouraging that the Afghanistan MCH handbook appeared to be valued and used by mothers across all socio economic and education levels.

## Conclusions

Our study showed that we have the potential to achieve almost universal coverage of a very basic but very essential MCH service in Afghanistan. WHO recommends that HBRs should be prioritised in remote, fragile settings with dynamic population movements [3]. Thus, our MCH handbook will be scaled up over the next three years across all of Afghanistan and our scale up will include close monitoring and assessment of coverage and use across all families especially poor families living in remote areas.

## Additional files

Additional file 1: Questionnaire used for data collection in the study. (PDF 975 kb)

Additional file 2: Characteristics of the women who lost their MCH handbook in Mirbachakot and Kama districts of Afghanistan from August 2017 to April 2018. (PDF 360 kb)

## Data Availability

The datasets used and/or analysed during the current study are available from the corresponding author on reasonable request.

## Abbreviations

ANC: Antenatal care
HBR: Home based record
MCH: Maternal and child health
PNC: Postnatal care
WHO: World Health Organization
