# Supplementary material for: Home-based records for poor mothers and children in Afghanistan, a cross sectional population based study
Source: BMC Public Health. 2019 Jun 17;19:766. doi: 10.1186/s12889-019-7076-7 (PMC6580634; doi:10.1186/s12889-019-7076-7)
Supplement: Supplementary file 1 — Questionnaire used for data collection in the study. (PDF 975 kb) [file 12889_2019_7076_MOESM1_ESM.pdf]

**Evaluation of the Implementation of Maternal and Child Health Handbook in Two Districts of Mir Bacha Kot in Kabul and Kama in Nangarhar**<br><br>**Targeting women who are eligible to receive a MCH HB.**		
	**INTERVIEW IDENTIFICATION**	
I1	Interviewer Code	|___|___|
I2	Day / Month<br>(auto-recorded)	|___ |___/ |___|___ |
I3	Time of Interview -- Start<br>(auto-recorded)	|___ |___| : |___|__ | AM PM
I4	Time of Interview – End<br>(auto-recorded)	|___ |___| : |___|__ | AM PM
I5	Province:	1. Kabul<br>2. Nangarhar
I6	District Name	1. Mir Bacha Kot<br>2. Kama
I7	Village Name	*Select from a list based on MoPH-provided*<br><br>*Will filter depending on health facility selected*
I9	Is the house vacated, destroyed or do neighbours say that the residents will be away for a substantial amount of time?<br>If yes, ends the survey	1. Yes<br>0. No
	**INTERVIEWER INTRODUCTION**	

INSTRUCTIONS:
(1) READ INFORMED CONSENT AND OBTAIN VERBAL CONSENT
(2) MUST COMPLETE ALL CODING BEFORE YOU LEAVE THE HOUSEHOLD

Guidance notes for introducing yourself and the purpose of the interview

Greet the interviewee according to the local culture of the area.

"Hello, My name is [*enumerator's name*] and I am working with a local research company. We are currently conducting research in collaboration with the Ministry of Public Health and UNICEF to better understand a new initiative call the MCH Handbook. Today I am looking to speak with a mother who has one or more children who is younger than 6 months. Is there currently a woman in this household who fits this description?
I want to assure you that all information collected today is completely confidential and no personal information will be shared. The answer provided today will be compiled with the results of other interviewees. Furthermore, your participation is completely voluntary and at any time should you not wish to continue, please let us know."

May I continue with the interview?
1) Yes
0)No……….End interview and replace the household

A1	Beneficiary Present: Is there a female that lives in this household meeting the following criteria:<br>• Married between the ages of 15 and 49<br>• Has had a child that is 6 months or less<br>• Lives in the household permanently, -not visiting a household as a guest<br>*(If the respondent answers "No," or "Refuse" end the survey and replace the household)*	1. Yes<br>0. No<br>99. Refuse
A2	I would like to speak to an eligible woman-is this OK?<br>*(If the respondent answers "No," or "Refuse" end the survey and replace the household)*	1. Yes<br>0. No
A3	Does this household meet the eligibility criteria?<br>(Only shown if answer to A1 or A2 are no/refuse)	1. Yes<br>1. No

	MODULE 1: RESPONDENT SELECTION	
**Interviewer note**	**Write down the name of every woman living in the household who meets the following criteria:**<br>• **Married**<br>• **Between the age of 15 and 49**<br>• **Has had a child born in the past 6 months**<br>• **Lives permanently in the household (not visiting or living as a guest)**<br><br>**This list should include ALL women who meet these eligibility criteria living in the household, even if they are not home when you visit the household.**<br><br>**Once all women are listed, put pieces of paper equal to the total number of eligible women in a hat/bowl (i.e. if there are 5 eligible women, you should put 5 pieces of paper in the hat/bowl). One piece of paper should have an 'X' written on it. Whoever draws the piece of paper with the 'X' will be interviewed.**<br><br>**If an eligible woman is not present and no woman present draws the paper with an 'X' on the first round, you must go back to the household when the eligible woman is around to survey her.**	
HL1	HOW MANY ELIGIBLE WOMEN LIVE IN THIS HOUSEHOLD?	|___|___|
HL2	Name of the randomly selected respondent	NAME OF SELECTED INTERVIEWEE<br>____
HL3	Age of Respondent (years)	

CHILD SELECTION AND CONFIRMATION		
CH1	HOW MANY TOTAL CHILDREN DO YOU HAVE?	(number)
CH2	HOW MANY MONTHS OLD IS YOUR YOUNGEST CHILD?	(number)
CH3	WHEN WAS YOUR CHILD BORN?<br><br>(IF ANSWERS '1' END SURVEY)	1. BEFORE THE INDEPENDENCE DAY OF AFGHANISTAN (19 AUGUST)<br>2. BETWEEN THE INDEPENDENCE DAY OF AFGHANISTAN AND Eid ul Adha (Sept. 2-4)<br>3. BETWEEN Eid ul Adha (Sept. 2-4) AND Ashura-tenth of Moharam (Oct. 1)<br>4. BETWEEN Ashura-tenth of Moharam (Oct. 1) AND The Prophet's (pbuh) Birthday (Dec. 1)<br>5. BETWEEN The Prophet's (pbuh) Birthday (Dec. 1) AND Liberation Day (15 February)<br>6. AFTER Liberation Day (15 February)
CH4	IS YOU YOUNGEST CHILD A BOY OR GIRL?	1. MALE<br>0. FEMALE
**DEMOGRAPHICS**		
WB1	WHAT IS YOUR MARITAL STATUS?<br>DO NOT READ-SELECT ONE	1. Married<br>2. Divorced<br>3. Widowed<br>99. Refused
WB2	HOW OLD WERE YOU WHEN YOU WERE MARRIED? (WRITE IN AGE IN YEARS)	(NUMBER)
WB3	WHAT IS YOUR ETHNICITY?<br>*(DO NOT READ OUT LOUD, SELECT ONE)*	1. PASHTUN<br>2. Tajik<br>3. Hazara<br>4. Uzbek<br>5. Turkmen<br>6. Arab<br>7. Baloch<br>8. Aimak<br>9. Sadat<br>10. Nuristani<br>11. Kuchi (nomads)<br>12. Other<br>99. REFUSED
WB4	WHAT IS THE HIGHEST LEVEL OF SCHOOL YOU COMPLETED?<br>*(DO NOT READ OUT LOUD, SELECT ONE)*	1. NO SCHOOL<br>2. PRIMARY (1-6)<br>3. SECONDARY (7-9)<br>4. HIGH SCHOOL (10-12)<br>5. Bachelors degree<br>6. Masters degree<br>7. Madrasa<br>8. Other (specify)<br>99. Refuse

Enumerator instructions: Show respondent your tablet and ask "Are you able to read the following sentence for me?"		
WB5	DO YOU KNOW HOW TO READ? *(do not ask- enumerator fills in based on performance of respondent)*	1. YES, can read whole sentence<br>2. YES, can read part of the sentence<br>3. NO, cannot read at all<br>99. REFUSE (*respondent refuses to participate in this question)*
WB6	DO YOU KNOW HOW TO WRITE?	1. YES<br>0. NO<br>99. REFUSE (DO NOT READ OUT LOUD)
WB7	WHAT IS/WAS YOUR HUSBAND'S HIGHEST LEVEL OF EDUCATION	1. NO SCHOOL<br>2. PRIMARY (1-6)<br>3. SECONDARY (7-9)<br>4. HIGH SCHOOL (10-12)<br>5. Bachelors degree<br>6. Masters degree<br>7. Madrasa<br>8. Other (specify)<br>99. Refuse
**SOCIO-ECONOMIC ASSESSMENT**		
INTERVIEWER GUIDE: (READ OUT LOUD) "I would now like to ask some questions about your household possessions. I hope this is not too sensitive but it is important for this study. Please know that there will be no financial incentive for answering these questions. All of the information you provide on this and all other parts of the survey will be kept confidential."<br><br>*These questions pertain to everyone in your household. A household includes ALL people living in the same social unit composed of people living together in the same dwelling permanently--adults, children, non-relatives living inside the dwelling)*		
SE1	HOW MANY SLEEPING ROOMS DO YOU HAVE IN YOUR HOME	|___|___| <br>99. REFUSE (*Do not read out loud)*
SE2	DOES YOUR HOUSEHOLD OWN A BICYCLE?	1. YES<br>0. NO<br>99. REFUSE
SE3	DOES YOUR HOUSEHOLD OWN A MOTORCYCLE?	1. YES<br>0. NO<br>99. REFUSE
SE4	DOES YOUR HOUSEHOLD OWN ANIMAL-DRAWN-CART?	1. YES<br>0. NO<br>99. REFUSE
SE5	DOES YOUR HOUSEHOLD OWN CAR/TRUCK?	1. YES<br>0. NO<br>99. REFUSE

SE6	DOES YOUR HOUSEHOLD OWN RADIO?	1. YES<br>0. NO<br>99. REFUSE
SE7	DOES YOUR HOUSEHOLD OWN TELEVISION?	1. YES<br>0. NO<br>99. REFUSE
SE8	DOES YOUR HOUSEHOLD OWN MOBILE PHONE?	1. YES<br>0. NO<br>99. REFUSE
SE9	DOES THIS HOUSEHOLD OWN ANY LIVESTOCK, HERDS, FARM ANIMALS, OR POULTRY?	1. YES<br>0. NO<br>99. REFUSE
SE10	HOW MANY OF THE FOLLOWING ANIMALS DOES THIS HOUSEHOLD HAVE?<br><br>***If none, record '00'.***<br>*IF UNKNOWN, RECORD '888'.*<br>*IF REFUSE '999'*	**[SES 10A]** MILK COWS AND/OR BULLS? _______<br><br>**[SES 10B]** HORSES AND/OR CAMELS?_______<br><br>**[SES 10c]** DONKEYS AND/OR MULES? ________<br><br>**[SES 10D]** SHEEP AND/OR GOATS? ________<br><br>**[SES 10E]** CHICKENS AND/OR DUCKS? ________
SE11	HOW MANY JERIBS OF LAND DOES THIS HOUSEHOLD OWN?<br><br>***If none, record '00'.***<br>*IF UNKNOWN, RECORD '888'.*<br>*IF REFUSE '999'*	**SE11A**______________jerib<br><br>**SE11B**_____________ beswa (1/20th of a jerib)
SE12	What kind of toilet facility do members of your household use?<br><br>DO NOT READ	1. VENTILATED IMPROVED PIT LATRINE<br>2. PIT LATRINE WITH SLAB<br>3. PIT LATRINE WITHOUT SLAB/OPEN PIT<br>4. COMPOSTING TOILET<br>5. BUCKET TOILET<br>6. TRADITIONAL VALUT TOILET<br>7. ECO SANITATION<br>8. NO FACILITY/BUSH/FIELDF<br>9. FLUSH OR POUR FLUSH TOILET/FLUSH TO PIPED SEWER SYSTEM<br>10. FLUSH TO SEPTIC TANK<br>11. FLUSH TO PIT LATRINE<br>12. FLUSH TO SOMEWHERE ELSE

		13. FLUSH-DON'T KNOW WHERE<br>14. Other<br>99. REFUSE
SE13.	WHAT IS YOUR PRIMARY SOURCE OF WATER?<br><br>DO NOT READ	1. HAND PUMP/PIPED WATER WITHIN HOME<br>2. PUBLIC TAP/STANDPIPE/HAND PIPE<br>3. PROTECTED DUG WELL<br>4. UNPROTECTED WELL<br>5. TANKER-TRUCK<br>6. UNPROTECTED SPRING/HAWZ<br>7. PROTECTED SPRING<br>8. SURFACE WATER (STREAM/RIVER/LAKE/KAREZ)<br>9. RAINWATER<br>10. BOTTLED WATER<br>11. TUBE WELL OR BOREHOLE<br>12. CART WITH SMALL TANK/DRUM<br>13. OTHER (SPECIFY) _______________<br>99. REFUSE
SE14.	WHAT IS THE MAIN MATERIAL OF EXTERIOR WALLS<br><br>DO NOT READ -- RECORD OBSERVATION	1. NO WALLS<br>2. CARDBOARD/TENT<br>3. DIRT WALL<br>4. HAY WITH MUD/STONE WITH MUD/PLYWOOD/REUSED WOOD<br>5. CEMENT<br>6. BRICKS<br>7. WOOD PLANKS/SHINGLES<br>8. OTHER (SPECIFY)_______________
SE15	**MAIN** MATERIAL OF THE ROOF.<br><br>DO NOT READ -- RECORD OBSERVATION	1. NO ROOF<br>2. NATURAL ROOFING: EARTH/SAND (GHORAGEL)/GRASS/SOD/MUD WITH GRASS<br>3. RUDIMENTARY ROOFING: RUSTIC MAT/MUD AND HAY/WOOD PLANK<br>4. CARDBOARD/TENT<br>5. FINISHED ROOFING: METAL/WOOD/CEMENT FIBER/CERAMIC TILES/CEMENT/ROOFING SHINGLES<br>6. OTHER (SPECIFY) _____________
SE16	WHAT IS THE MAIN MATERIAL OF THE FLOOR (OBSERVE, DO NOT ASK)	1. NATURAL FLOOR (EARTH/SAND/DUNG/MUD AND HAY<br>2. RUDIMENTARY FLOORS (WOOD PLANKS)<br>3. FINISHED FLOORS (PARQUE OR POLICED WOOD/VINYL OR ASPHALT STRIPS/CERAMIC TILE/CEMENT/RUGS/MAT/CARPET

		4. OTHER (SPECIFY)
ENUMERATOR INSTRUCTIONS (READ OUT LOUD)-I would now like to ask you some questions about how to care for yourself and your baby both before and after birth.		
KNOWLEDGE		
K1	HOW MANY TIMES SHOULD A PREGNANT WOMAN SEE A HEALTH CARE PROVIDER FOR ANTENATAL CARE? (DO NOT READ OUT LOUD, SELECT 1 RESPONSE)	1. 1<br>2. 2<br>3. 3<br>4. 4 OR MORE<br>98. DON'T KNOW<br>99. REFUSE
K2	WHAT CARE SHOULD YOU RECEIVE AS PART OF AN ANC VISIT WITH A HEALTH PROVIDER (DO NOT READ OUT LOUD, CIRCLE ALL THAT APPLY)	K2_1 HEIGHT MEASUREMENT<br>1. YES<br>0. NO
		K2_2 WEIGHT MEASUREMENT FOR WEIGHT GAIN<br>1. YES<br>0. NO
		K2_3 BLOOD RESSURE TEST<br>1. YES<br>0. NO
		K2_4 IRON FOLIC ACID TABLETS (TABLET TO PREVENT ANEMIA AND NEONATAL MALFORMATION)<br>1. YES<br>0. NO
		K2_5 TETANUS TOXOID INJECTION/INJECTION TO PREVENT TETANUS, THAT IS CONVULSTIONS AFTER BIRTH<br>1. YES<br>0. NO
		K2_6 CALCIUM TABLETS<br>1. YES<br>0. NO
		K2_8OTHER<br>1. YES<br>0. NO
		K2_98. DON'T KNOW<br>1. YES

		0. NO
		K2_99. REFUSE<br>1. YES<br>0. NO
K3	WHAT ARE SOME DANGER SIGNS DURING PREGNANCY? (DO NOT READ OUT LOUD, CIRCLE ALL THAT APPLY)	K3_1. BLEEDING<br>1. YES<br>0. NO
		K3_2. HIGH FEVER<br>1. YES<br>0. NO
		K3_3. CONVULSION, SEVERE HEADACHE/BLURRED VISION<br>1. YES<br>0. NO
		K3_4. EDEMA OF FEET, HANDS OR FACE<br>1. YES<br>0. NO
		K3_5. ABDOMINAL PAIN AND/OR FLUID LEAKAGE BEFORE THE DUE DATE<br>1. YES<br>0. NO
		K3_6. Other [write in] _____________________<br>1. YES<br>0. NO
		K3_98. DON'T KNOW<br>1. YES<br>0. NO
		K3_99. REFUSE<br>1. YES<br>0. NO
K4	HOW MANY TIMES SHOULD A PREGNANT WOMAN SEE A HEALTH CARE PROVIDER FOR CARE AFTER BIRTH, ALSO CALLED POSTNATAL CARE?	1. 1<br>2. 2<br>3. 3<br>4. 4 OR MORE<br>98. DON'T KNOW<br>99. REFUSE

	(DO NOT READ OUT LOUD, SEELCT 1 RESPONSE)	
K5	WHEN SHOULD YOUR CHILD RECEIVE YOUR FIRST DOSE OF THE OPV IMMUNIZATION? (DO NOT READ OUT LOUD, SELECT 1 RESPONSE)	1. BEFORE THE BABY IS BORN<br>2. DIRECTLY AFTER BIRTH<br>3. WHEN THE CHILD IS 3-6 MONTHS OLD<br>4. WHEN THE CHILD IS 7 MONTHS OR OLDER<br>5. THE CHILD DOES NOT NEED AN OPV VACCINATION<br>98. DON'T KNOW<br>99. REFUSE
K6	HOW OFTEN SHOULD YOU CHECK YOUR CHILD'S GROWTH?	1. YOU DO NOT NEED TO MONITOR YOUR CHILDS GROWTH<br>2. ONCE A YEAR<br>3. RIGHT AFTER BIRTH ONLY<br>4. EVERY MONTH FOR 1 YEAR<br>5. EVERY MONTH FOR 2 YEARS<br>98. DO NOT KNOW<br>99. REFUSE
K7	WHEN SHOULD A WOMAN START BREASTFEEDING HER BABY?<br>(DO NOT READ OUT LOUD, SEELCT 1 RESPONSE)	1. IT IS NOT NECESSARY TO BREASFEED<br>2. WITHIN 1 HOUR AFTER BIRTH<br>3. WITHIN 24 HOURS (1 DAY) AFTER BIRTH<br>5. OTHER (SPECIFY)<br>98. DON'T KNOW<br>99. REFUSE
**RETENTION OF RECORDS**		
R1	HAVE YOU EVEN BEEN GIVEN A RECORD—BOOKLET, CARD, OR PAPER—RELATED TO YOUR CHILD'S HEALTH?	1. YES<br>0. NO<br>99. REFUSE
R2	I AM GOING TO READ A LIST OF MATERIALS USED TO RECORD HEALTH SERVICES FOR MOTHER AND BABY. PLEASE TELL ME IF YOU RECEIVED THE CARD, RECEIVED BUT LOST/NO LONGER HAVE THE CARD, OR NEVER RECEIVED THIS. (READ EACH OUT LOUD. MAY SELECT MULTILE)	R2_1. MCH HANDBOOK<br>1. YES<br>2. LOST THE CARD<br>3. NEVER RECEIVED<br>99. REFUSED
		R2_2. VACCINATION CARD<br>1. YES<br>2. LOST THE CARD<br>3.. NEVER RECIEVED<br>99. REFUSED
		R2_3. ANC CARD<br>1. YES<br>2. LOST THE CARD<br>3.. NEVER RECIEVED

	IF 'YES' TO MCH-HB SKIP TO R8	99. REFUSED
		R2_4. PNC CARD<br>1. YES<br>2. LOST THE CARD<br>3.. NEVER RECIEVED<br>99. REFUSED
		R2_5. FAMILY PLANNING CARD<br>1. YES<br>2. LOST THE CARD<br>3.. NEVER RECIEVED<br>99. REFUSED
		R2_6. OTHER (SPECIFY)<br>1. YES<br>2. LOST THE CARD<br>3.. NEVER RECIEVED<br>99. REFUSED
R3	PLEASE TELL ME WHICH YOU HAVE RECEIVED (DO NOT READ, CIRCLE ALL THAT ARE PROVIDED)<br><br>ENUMERATOR NOTE: WRITE THE DATE OF RECEIPT AS NOTED ON THE CARD. IF THEY HAVE LOST THE CARD, YOU SHOULD WRITE AN APPROXIMATE DATE. IF NO APPROXIMATE DATE IS AVAILABLE, SELECT '98'	R3_1_date. MCH handbook<br>*If yes: approximate date/year*<br>*98. Don't know*
		R3_2_date. Vaccination card<br>*If yes: approximate date/year*<br>*98. Don't know*
		R3_3_date. Antenatal card<br>*If yes: approximate date/year*<br>*98. Don't know*
		R3_4_date. Postnatal card<br>*If yes: approximate date/year*<br>*98. Don't know*
		R3_5_date Family planning card<br>*If yes: approximate date/year*<br>*98. Don't know*
		R3_6_date. Other (specify)<br>*If yes: approximate date/year*<br>*98. Don't know*
R4	IF RESPONDENT SAYS THEY RECEIVED BUT LOST THEIR MCH HANDBOOK: WHAT COLOR WAS THE HANDBOOK?*(DO NOT READ OUT*	1. BLUE<br>2. YELLOW<br>3. PINK/RED<br>4. GREEN<br>98. DON'T KNOW<br>99. REFUSE

	LOUD-SELECT ONE ANSWER)<br><br>ONLY ANSWER IF RESPONDENT SAID 'LOST THE CARD' FOR R2-A	
R5	IF RESPNDENT SAID THEY RECEIVED BUT LOST THEIR MCH HANDBOOK: WHAT INFORMATION WAS RECORDED IN THE HANDBOOK? (DO NOT READ OUT LOUD-CIRCLE ALL THAT APPLY)<br><br>ONLY ANSWER IF RESPONDENT SAID 'LOST THE CARD' FOR R2_1	R5_1. NUMBER OF ANC VISITS<br>1. YES<br>0. NO
		R5_2. NUMBER OF PNC VISITS<br>1. YES<br>0. NO
		R5_3. POST PARTUM FAMILY PLANNING CARD/COMMON FAMILY PLANNING CARD<br>1. YES<br>0. NO
		R5_4. DELIVERY record<br>1. YES<br>0. NO
		R5_5. MEDICAL HISTORY<br>1. YES<br>0. NO
		R5_Child Immunization<br>1. YES<br>0. NO
		R5_7. Growth monitoring<br>1. YES<br>0. NO
		R5_8. Others (specify..............................)<br>1. YES<br>0. NO
		R5_98. Don't know<br>1. YES<br>0. NO
		R5_99. Refuse<br>1. YES<br>0. NO

R6	IF RESPONDENT SAID THEY HAD NEVER RECEIVED A HANDBOOK SHOW THEM A PICTURE OF THE HANDBOOK AND ASK: HAVE YOU EVER SEEN THIS BOOK?<br><br>ONLY ANSWER IF RESPONDENT SAID 'NEVER RECEIVED' FOR R2A	1. YES<br>0. NO<br>99. REFUSE TO ANSWER
R7	IF THE RESPONDENT SAYS YES: WHAT IS THIS BOOK FOR? (DO NOT READ OUT LOUD, CIRCLE ALL THAT APPLY)<br>ONLY ANSWER IF RESPONDENT SAID 'NEVER RECEIVED' FOR R2A	R7_1. FOR MOTHER AND CHILD HEALTH<br>1. YES<br>0. NO
		R7_2. TO KEEP HEALTH RECORDS<br>1. YES<br>0. NO
		R7_3. OTHER (SPECIFY)<br>1. YES<br>0. NO
		R7_98. Don't know<br>1. YES<br>0. NO
		R7_99. Refuse<br>1. YES<br>0. NO
R8	WHO PROVIDES THESE BOOKS?<br>(DO NOT READ OUT LOUD, CIRCLE ALL THAT APPLY)<br>ONLY ANSWER IF RESPONDENT SAID 'NEVER RECEIVED' FOR R2A	R8_1. COMMUNITY LEADERS<br>1. YES<br>0. NO
		R8_2. PUBLIC HEALTH CENTERS (CHC, BHC, DH)<br>1. YES<br>0. NO
		R8_3. COMMUNITY HEALTH WORKERS<br>1. YES<br>0. NO
		R8_4. RELIGIOUS LEADERS<br>1. YES

		0. NO
		R8_5. PRIVATE HEALTH CENTERS<br>1. YES<br>0. NO
		R8_6. OTHER (specify)<br>1. YES<br>0. NO
		R8_98. DON'T KNOW<br>1. YES<br>0. NO
		R8_99.REFUSE<br>1. YES<br>0. NO
INTERVIEWER GUIDE: (READ OUT LOUD) "I would now like to ask some questions about your maternal health seeking behaviour. For all of these questions, please be specific about your health services received during, before, and after your child that is aged 0-6 months. All of the questions below refer to this child.<br><br>*(A household includes ALL people living in the same social unit composed of people living together in the same dwelling--adults, children, non-relatives living inside the dwelling)*		
MNC1	FOR YOUR YOUNGEST CHILD (0-6 MONTHS) DID YOU RECEIVE ANY ADVICE BEFORE DELIVERY, ALSO CALLED ANTENATAL CARE?<br><br>IF NO, GO TO MNC4	0. NO<br>1. YES – the data from MCH HB<br>2. YES- the data from any cards/handbook other than MCH Handbook<br>3. YES – the recall of the respondent<br>99. REFUSE
MNC2	FROM WHOM DID YOU RECEIVE YOUR ANC? (FOR YOUR YOUNGEST CHILD 0-6 MONTHS)<br>**DO NOT READ OUT LOUD**<br><br>**MAY SELECT MULTIPLE**<br><br>IF DOES NOT SELECT OPTION 1, 2, 3 OR 4 THEN SKIP TO MNC4	Mnc2_1 DOCTOR AT A PUBLIC HEALTH CENTER<br>1. YES<br>0. NO
		Mnc2_2 DOCTOR AT A PRIVATE HEALTH CENTER<br>1. YES<br>0. NO
		Mnc2_3 NURSE/MIDWIFE AT A PUBLIC HEALTH CENTER<br>1. YES<br>0. NO
		Mnc2_4 NURSE/MIDWIFE AT A PRIVATE HEALTH CENTER<br>1. YES<br>0. NO

		Mnc2_5 COMMUNITY MIDWIFE<br>1. YES<br>0. NO
		Mnc2_6 TRADITIONAL BIRTH ATTENDANT<br>1. YES<br>0. NO
		Mnc2_7 COMMUNITY HEALTH WORKER<br>1. YES<br>0. NO
		Mnc2_8 OTHER (SPECIFY)<br>1. YES<br>0. NO
		Mnc2_9 health clinic (unspecified service provider)<br>1. YES<br>0. NO
		Mnc2_99. REFUSE<br>1. YES<br>0. NO
MNC3	HOW MANY ANC VISITS DID YOU MAKE FROM HEALTH SERVICE PROFESSIONALS (DOCTORS, NURSES OR MIDWIVES) DURING YOUR LAST PREGNANCY FOR YOUR YOUNGEST CHILD (0-6 MONTHS)<br><br>THIS DOES **NOT** INCLUDE INFORMATION/ADVICE PROVIDED BY CHWS, FAMILY/FRIENDS, TBAS<br><br>*DO NOT READ OUT LOUD*<br><br>*MARK ONLY ONE ANSWER*	1. 1<br>2. 2<br>3. 3<br>4. 4<br>5. More than 4 (Specify) _______________<br>97. NOT ASKED (NO ANC VISITS)<br>99. REFUSE
MNC3_MORE	HOW MANY ANC VISITS DID YOU RECEIVE? (ONLY IF ANSWERED '5')	Number—more than 4
MNC4	WHERE DID YOU GIVE BIRTH TO YOUR YOUNGEST CHILD (0-6 MONTHS)<br>*DO NOT READ OUT LOUD*	1. PUBLIC PROVINCIAL HOSPITAL<br>2. PUBLIC DISTRICT HOSPITAL<br>3. PUBLIC CHC<br>4. PUBLIC BHC

	MARK ONLY ONE ANSWER	5. PUBLIC HEALTH SUB-CENTER<br>6. AT HOME WITH RELATIVE/FRIEND<br>7. AT HOME WITH TRADITIONAL BIRTH ATTENDANT<br>8. AT HOME WITH TRAINED MIDWIFE/SKILLED BIRTH ATTENDANT<br>9. AT HOME OR IN A HEALTH-POST WITH A CHW<br>10. PUBLIC HEALTH POST<br>11. MOBILE HEALTH TEAM<br>12. PRIVATE HEALTH FACILITY<br>13. OTHER<br>14. CLINIC UNSPECIFIED<br>99. REFUSE
MNC5	IN YOUR LAST PREGNANCY FOR YOUR YOUNGEST CHILD (0-6 MONTHS)-DID YOU RECEIVE ADVICE OR SERVICES AFTER DELIVERY, ALSO CALLED POST-NATAL CARE?<br><br>NOTE IF RESPONDENT HAS CARD, ASK TO SEE IT<br><br>**IF NO OR "REFUSE," MARK SKIP TO MNC8**	0. NO<br>1. YES – the data from MCH HANDBOOK<br>2. YES – the data from other than MCH HANDBOOk<br>3. YES- the recall of the respondent<br>99. REFUSE
MNC6	FROM WHOM DID YOU RECEIVE YOUR MAIN SOURCE OF PNC FOR YOUR YOUNGEST CHILD (0-6 MONTHS) ?<br>**DO NOT READ OUT LOUD**<br><br>**MAY SELECT MULTIPLE**<br><br>IF DOES NOT SELECT OPTION '1' OR '2', '3' OF '4' SKIP TO MNC8	Mnc6_1 DOCTOR AT A PUBLIC HEALTH CENTER<br>1. YES<br>0. NO
		Mnc6_2 DOCTOR AT A PRIVATE HEALTH CENTER<br>1. YES<br>0. NO
		Mnc6_3 NURSE/MIDWIFE AT A PUBLIC HEALTH CENTER<br>1. YES<br>0. NO
		Mnc6_4 NURSE/MIDWIFE AT A PRIVATE HEALTH CENTER<br>1. YES<br>0. NO
		Mnc6_5 COMMUNITY MIDWIFE<br>1. YES<br>0. NO
		Mnc6_6 TRADITIONAL BIRTH ATTENDANT<br>1. YES<br>0. NO

		Mnc6_7 COMMUNITY HEALTH WORKER<br>1. YES<br>0. NO
		Mnc6_8 OTHER (SPECIFY)<br>1. YES<br>0. NO
		Mnc6_9 Health clinic (unspecified provider)<br>1. YES<br>0. NO
		Mnc6_99. REFUSE<br>1. YES<br>0. NO
**MNC7**	HOW MANY TIMES DID YOU GO TO A HEALTH CENTRE TO GET PNC FOR YOUR YOUNGEST CHILD (0-6 MONTHS) ?<br><br>THIS DOES **NOT** INCLUDE INFORMATION/ADVICE PROVIDED BY CHWS, FAMILY/FRIENDS, TBAS<br><br>**DO NOT READ OUT LOUD**<br><br>**MARK ONLY ONE ANSWER**	1. 1<br>2. 2<br>3. 3<br>4. 4<br>5. More than 4 (SPECIFY) __________<br>97. NOT ASKED (NO PNC VISITS)<br>99. REFUSE
**MNC7_MORE**	HOW MANY PNC VISITS DID YOU RECEIVE? (ONLY IF SELECTED '5')	Number greater than 5
**MNC8**	DID YOU RECEIVE A PENTA1 VACCINATION FOR YOUR YOUNGEST CHILD (0-6 MONTHS) ?	0. NO<br>1. YES – the data from MCH HB<br>2. YES- the data from any cards/handbook other than MCH Handbook<br>3. YES – the recall of the respondent<br>98. DON'T KNOW<br>99. REFUSE
**MNC9**	DID YOUR CHILD RECEIVE AN OPV VACCINATION FOR YOUR YOUNGEST CHILD (0-6 MONTHS?<br><br>IF NO, 'DON'T KNOW' OR 'REFUSE' SKIP TO MNC11	0. NO<br>1. YES – the data from MCH HB<br>2. YES- the data from any cards/handbook other than MCH Handbook<br>3. YES – the recall of the respondent<br>98. DON'T KNOW<br>99. REFUSE

**MNC10**	IF YES, WHEN WAS THEIR FIRST DOSE?	1. RIGHT AFTER BIRTH<br>2. 6 WEEKS AFTER BIRTH<br>3. 10 WEEKS AFTER BIRTH<br>4. 14 WEEKS AFTER BIRTH<br>5. AT 9 MONTHS<br>6. AT 18 MONTHS<br>99. REFUSE
**MNC11**	WAS YOUR CHILD'S GROWTH MONITORED DURING VISITS TO A HEALTH CENTER?	0. NO<br>1. YES – the data from MCH HB<br>2. YES- the data from any cards/handbook other than MCH Handbook<br>3. YES – the recall of the respondent<br>98. DON'T KNOW<br>99. REFUSE
**Enumerator instructions: I am not going to ask you a series of questions about the maternal and child health handbook. For each question I am only asking about the handbook you received for your child who is now 0-6 months. I do not need to know about handbooks you received for other children or for yourself if you are currently pregnant.**		
R9	IF RESPONDENT RECEIVED A MCH HB: HOW MANY MCH HANDBOOKS DID YOU RECEIVE FOR YOUR LAST CHILD (CHILD AGED 0-6)<br><br>DO NOT READ OUT LOUD, CIRCLE 1	0. Did not receive a handbook<br>1. 1<br>2. 2<br>3. 3 or more (specify)<br>98. Don't know<br>99. Refused
R10	FROM WHERE DID YOU RECEIVE A MCH HANDBOOK (IF RECEIVED ONLY ONE, ONLY ONE RESPONSE IS ALLOWED, IF RECEIVED 2-TWO ANSWERS ALLOWED, IF RECEIVED 3 OR MORE, 3 ANSWERS ALLOWED)<br><br>DO NOT READ OUT LOUD	R10_kama_1. If in Kama:<br>1. Kama District Hospital<br>2. Sangar Sarai Comprehensive Health Center<br>3. Landaboch Basic Health Center<br>4. Zakhel Basic Health Center<br>5. CHW in community<br>6. Other (specify)<br>99. Refuse<br>R10_mbc_1. If in Mir Bacha Kot:<br>1. Mirbacha Kot District Hospital<br>2. Shaikhan Basic Health Center<br>3. Dakoo Basic Health Center<br>4. Gozar Basic Health Center<br>5. CHW in community<br>6. Other (specify)<br>99. Refuse

	FROM WHERE DID YOU RECEIVE YOUR SECOND HANDBOOK?<br>(ONLY IF SELECTED '2' OR '3' FOR R9)	R10_kama_1. If in Kama:<br>7. Kama District Hospital<br>8. Sangar Sarai Comprehensive Health Center<br>9. Landaboch Basic Health Center<br>10. Zakhel Basic Health Center<br>11. CHW in community<br>12. Other (specify)<br>99. Refuse<br>R10_mbc_1. If in Mir Bacha Kot:<br>7. Mirbacha Kot District Hospital<br>8. Shaikhan Basic Health Center<br>9. Dakoo Basic Health Center<br>10. Gozar Basic Health Center<br>11. CHW in community<br>12. Other (specify)<br>99. Refuse
	FROM WHERE DID YOU RECEIVE YOUR SECOND HANDBOOK?<br>(ONLY IF SELECTED '2' OR '3' FOR R9)	R10_kama_2. If in Kama:<br>13. Kama District Hospital<br>14. Sangar Sarai Comprehensive Health Center<br>15. Landaboch Basic Health Center<br>16. Zakhel Basic Health Center<br>17. CHW in community<br>18. Other (specify)<br>99. Refuse<br>R10_mbc_2. If in Mir Bacha Kot:<br>13. Mirbacha Kot District Hospital<br>14. Shaikhan Basic Health Center<br>15. Dakoo Basic Health Center<br>16. Gozar Basic Health Center<br>17. CHW in community<br>18. Other (specify)<br>99. Refuse
R11	WHEN YOU RECEIVED THE HB-WERE YOU TOLD ANYTHING ABOUT IT?<br><br>DO NOT READ OUT LOUD, MAY SELECT MULTIPLE	R11_1. NO INFORMATION PROVIDED<br>1. YES<br>0. NO
		R11_2. IT KEEPS RECORDS OF YOUR CHILDREN'S HEALTH CARE<br>1. YES

		0. NO
		R11_3. IT SHOULD BE KEPT<br>1. YES<br>0. NO
		R11_4. YOU SHOULD SHOW IT TO THE HEALTH WORKER FOR EACH VISIT FOR YOUR CHILD<br>1. YES<br>0. NO
		R11_5. IT CONTAINS USEFUL INFORMATION ON HOW TO CARE FOR A CHILD (HEALTH PROMOTION MESSAGES)<br>1. YES<br>0. NO
		R11_6. OTHER (SPECIFY)<br>1. YES<br>0. NO
		R11_99. REFUSE<br>1. YES<br>0. NO
R12	PLEASE TELL ME HOW YOU HAVE USED THE HANDBOOK	
	R12_A. READ THE MESSAGES	1. YES<br>0. NO<br>99. REFUSE
	R12_B. REVIEW YOUR AND YOUR CHILD'S HEALTH RECORDS	1. YES<br>0. NO<br>99. REFUSE
	R12_C. SHOW TO OTHER FAMILY MEMBERS?	1. YES<br>0. NO<br>99. REFUSE
	R12_D. SHOW TO FRIENDS OR NEIGHBORS	1. YES<br>0. NO<br>99. REFUSE
	R12_E. TAKE IT TO VISITS WITH HEALTH WORKERS?	1. YES<br>0. NO<br>99. REFUSE
	R12_F. LOOKED AT THE	1. YES

	ILLUSTRATIONS?	0. NO<br>99. REFUSE
R13	I AM GOING TO READ A LIST OF 10 PARTS OF THE MCH HB-PLEASE TELL ME THE TOP THREE THINGS THAT WERE USEFUL TO YOU? SELECT THREE	
	R13_1	WRITTEN INFORMATION ON anc
	R13_2	written information on keeping mother health
	R13_3	written information on care after the baby is born
	R13_4	illustrations
	R13_5	vaccination records,
	R13_6	growth charts
	R13_7	PNC Records
	R13_8	ANC Records
	R13_9	written information on child-health
	R13_10	continuum of care table/summary of care flowers at end
	R13_11	Other
**COMPLETENESS OF HEALTH RECORD**		
C1	NAME RECORDED	1. YES AND LEGIBLE<br>2. YES AND NOT LEGIBLE<br>0. NO
C2	DATE OF BIRTH	1. YES AND LEGIBLE<br>2. YES AND NOT LEGIBLE<br>0. NO
C3	ANC	1. YES AND LEGIBLE<br>2. YES AND NOT LEGIBLE<br>0. NO
C3A	HOW MANY ANC VISITS RECORDED? (WRITE NUMBER)	
C4	PNC	1. YES AND LEGIBLE<br>2. YES AND NOT LEGIBLE<br>0. NO
C4A	HOW MANY PNC VISITS RECORDED (WRITE NUMBER)	
C5	BIRTH REGISTRATION?	1. YES AND LEGIBLE<br>2. YES AND NOT LEGIBLE<br>0. NO
C6	BIRTH DOSE OPV?	1. YES AND LEGIBLE<br>2. YES AND NOT LEGIBLE<br>0. NO
C7	OPV1?	1. YES AND LEGIBLE<br>2. YES AND NOT LEGIBLE<br>0. NO
C8	PENTA1?	1. YES AND LEGIBLE<br>2. YES AND NOT LEGIBLE<br>0. NO
C10	RECORD OF WEIGHT	1. YES AND LEGIBLE

	AFTER BIRTH?	2. YES AND NOT LEGIBLE<br>0. NO
C11	CONNECTION OF PLOTTING ON GROWTH CARD TO SHOW TRENDS IN GROWTH?	1. YES AND LEGIBLE<br>2. YES AND NOT LEGIBLE<br>0. NO
C12	ANY ILLEGIBLE ENTRIES FOR OTHER RECORDS?	1. YES AND LEGIBLE<br>2. YES AND NOT LEGIBLE<br>0. NO
C12_OTHER	WHICH RECORDS? (SPECIFY)	
**CONTACT SHEET**: ENUMERATOR INSTRUCTIONS: Thank you for your participation. It was very helpful and important for understanding and improving maternal health in Afghanistan. I would like to ask for a contact number if that is possible in case we need to ask for any clarification to your answers. It can be family member or other relative if that is better. II would like to say again that all of your information you shared with me today. All information you shared will be kept confidential. Thank you again!		
	Can you please provide a contact number for me to reach you?<br>*IF YOU CAN PROVIDE MORE THAN ONE THAT WOULD BE VERY GOOD*	Cont_1 Personal Number<br>___________________________________<br>Cont_2 Family Number<br>___________________________________<br>Cont_3 Relatives Number<br>___________________________________

Com	
Survey_time	Survey end time (auto record_
GPSpoints	GPS tag (auto recorded)
_GPSpoints_latitude	GPS latitude (auto recorded)
_GPSpoints_longitude	GPS longitude (autorecorded)
_GPSpoints_precision	GPS precision (auto recorded)
Meta/instanceID	identifer
_id	Unique id generated by platform (auto recorded)
_uuid	ID
_sumbission_time	Time data submitted/uploaded to server
