# Supplementary material for: Home-based records for poor mothers and children in Afghanistan, a cross sectional population based study
Source: BMC Public Health. 2019 Jun 17;19:766. doi: 10.1186/s12889-019-7076-7 (PMC6580634; doi:10.1186/s12889-019-7076-7)
Supplement: Supplementary file 2 — Characteristics of the women who lost their MCH handbook in Mirbachakot and Kama districts of Afghanistan from August 2017 to April 2018. (PDF 360 kb) [file 12889_2019_7076_MOESM2_ESM.pdf]

ADDITIONAL FILE 2

Additional file 2. Characteristics of the women who lost their MCH handbook in Mirbachakot and Kama districts of Afghanistan from August 2017 to April 2018

**Characteristic**		**Total (n=1,943)**	**Number of women who lost their handbook (n=10)**
Wealth quintile			
	Poorest (1)	361 (18.7%)	1
	2	361 (18.6%)	0
	3	361 (18.6%)	0
	4	361 (18.6%)	4
	Least poor (5)	360 (18.5%)	5
	Not known	139 (7.2%)	1
Maternal education			
	No education	1,524 (78.4%)	5
	Primary	149 (7.7%)	3
	Secondary+	170 (8.8%)	2
	Not known	100 (5.2%)	0
Maternal age			
	16-19y	74 (3.8%)	1
	20-24y	580 (29.9%)	4
	25-29y	540 (27.8%)	1
	30-34y	431 (22.2%)	1
	35+y	318 (16.4%)	3
Parity			
	1	304 (15.7%)	1
	2-6	1,290 (66.4%)	6
	7+	349 (18.0%)	3
Sex of youngest child			
	Female	941 (48.4%)	6
	Male	1,002 (51.6%)	4
District			
	Mirbachakot	849 (43.6%)	5
	Kama	1094 (56.3%)	5

MCH = maternal and child health
